# Clinical manifestations in a Chinese girl with heterozygous *de novo NAA10* variant c. 247C > T, p. (Arg83Cys): a case report

**DOI:** 10.3389/fped.2023.1198906

**Published:** 2023-06-27

**Authors:** Kaiyan Wei, Chaochun Zou

**Affiliations:** Department of Endocrinology, Children's Hospital, Zhejiang University School of Medicine, National Clinical Research Center for Child Health, Hangzhou, China

**Keywords:** NAA10, NAA10-related syndrome, n-terminal acetylation, ogden syndrome, case report

## Abstract

The *NAA10* gene encodes the catalytic subunit of the N-terminal acetyltransferase protein complex A (NatA), which is supposed to acetylate approximately 40% of the human proteins. After the advent of next-generation sequencing, more variants in the *NAA10* gene and Ogden syndrome (OMIM# 300855) have been reported. Individuals with *NAA10*-related syndrome have a wide spectrum of clinical manifestations and the genotype–phenotype correlation is still far from being confirmed. Here, we report a three years old Chinese girl carrying a heterozygous *de novo NAA10* [NM_003491: c. 247C > T, p. (Arg83Cys)] variant (dbSNP# rs387906701) (ClinVar# 208664) (OMIM# 300013.0010). The proband not only has some mild and common clinical manifestations, including dysmorphic features, developmental delay, obstructive hypertrophic cardiomyopathy, and arrhythmia, but also shows some rare clinical features such as exophthalmos, blue sclera, cutaneous capillary malformations, and adenoid hypertrophy. Our attempt is to expand the clinical phenotype associated with *NAA10*-related syndrome and explore genotype–phenotype correlation with such syndrome.

## Introduction

N-terminal acetylation (NTA), which is carried out by N-terminal acetyltransferases, is one of the most common protein modifications (1, 2). The N-terminal acetyltransferase protein complex A (NatA)is responsible for N-terminal acetylating approximately 40% of all human proteins and is composed of the catalytic subunit *NAA10* and the auxiliary subunits NAA15, NAA50, and HYPK (3, 4). The human *NAA10* gene, which encodes the catalytic subunit of the NatA, is an essential gene located in Xq28. Previous studies have shown that many hereditary or *de novo* NAA10 variants are pathogenic (5–7). Originally, a missense variant *NAA10* p. (Ser37Pro) was identified as the reason for an X-linked recessive lethal disorder in eight males from two families, which was called Ogden syndrome (OMIM# 300855) in 2011 (8). With the development of next-generation sequencing technology, a series of new *NAA10* variants have been reported, and the phenotypic spectrum of patients has broadened rapidly (6, 9–12). Although postnatal developmental retardation, intellectual disability (ID), and cardiac anomalies may be the main clinical manifestations, individuals exhibit heterogenous phenotypes with no clear genotype-phenotype correlation (5). Therefore, researchers proposed that this series of disorders should be referred to more broadly as *NAA10*-related syndrome (13).

Here, we report a heterozygous *de novo* variant of *NAA10* in a three-year-old Chinese girl whose whole exome sequencing (WES) identified a mutation of c. 247C > T, p. (Arg83Cys) (dbSNP# rs387906701) (ClinVar# 208664) (OMIM# 300013.0010). The girl showed developmental retardation, intellectual disability, and growth failure, which are common in *NAA10*-related syndrome. Meanwhile, she did exhibit some special clinical features and a less severe phenotype of cardiovascular defects, as well as recurrent respiratory tract infections. Our report further expands the mutation and clinical spectrum associated with *NAA10*-related syndrome and provides insight into its natural history and life trajectory, which contributes to the identification and comprehensive study of *NAA10*-related syndrome.

## Case presentation

Our patient was a girl who was the second child of healthy and non-consanguineous parents with Chinese origin. She was born naturally at 40 weeks of gestation. Her global exhibition was normal at birth, and her body weight and body length were 3,960 g and 50 cm, respectively. However, when she was 3 months old, the auscultation of the heart found a grade III murmur of puffing character. Then she was diagnosed with Hypertrophic Cardiomyopathy (HCM) by echocardiography.

**Figure 1:**
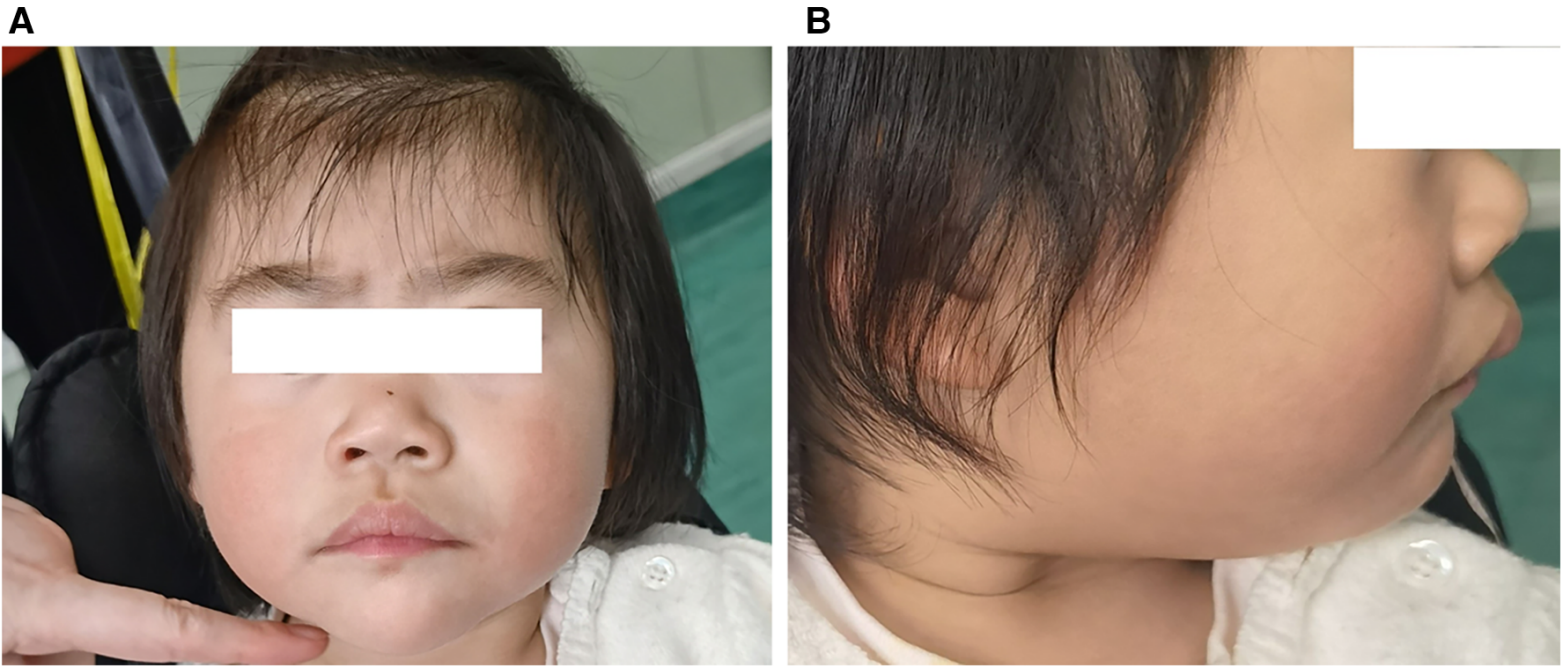
Clinical features of the patient at the age of 2 years: thick eyebrows, broad and flat nasal bridge, flared nares, short columella, wide philtrum, and protruding upper lip (**A**), large and low-set ears (**B**).

Several dysmorphic features were also found in the subsequent physical examination, including thick eyebrows, blue sclera, ocular hypotelorism, prominent eyes, large and low-set ears, broad and flat nasal bridge, flared nares, short columella, wide philtrum and mouth and protruding upper lip (Figures 1A,B). The skin was characterized by redundancy or laxity with cutaneous capillary malformations. While the developmental defects were not present in the extremities.

**Figure 2:**
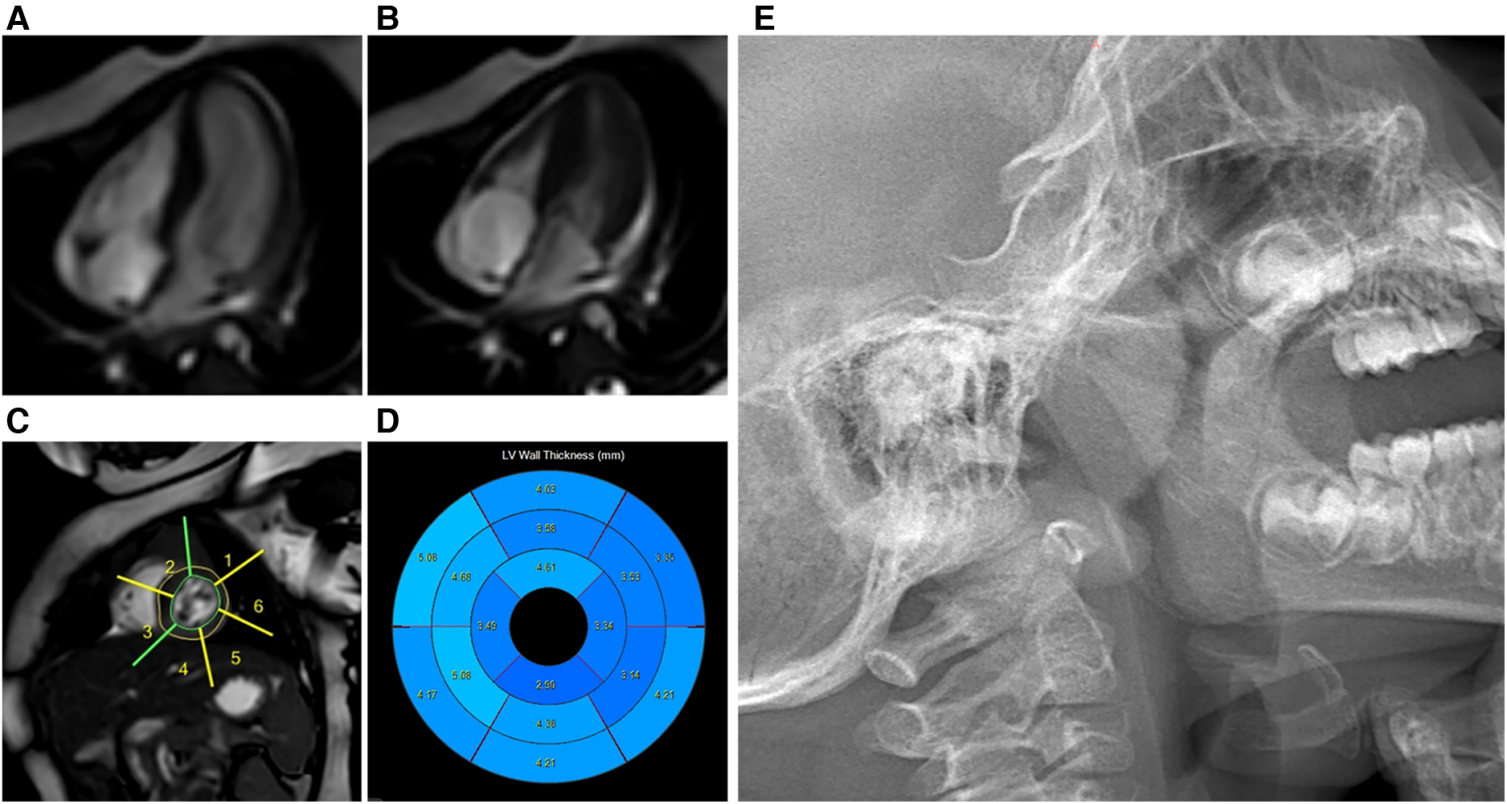
Cardiac MRI: obstructive hypertrophic cardiomyopathy with heterogeneous left ventricular hypertrophy. Cardiac magnetic resonance cine images from mid-ventricular short-axis and four-chamber views at end-diastole (**A**) and end-systole (**B**); Cardiac magnetic resonance cine images (**C**); Bulls-eye plot representing the thickness of the left ventricle (**D**); Lateral nasopharyngeal x-ray: adenoidal hypertrophy and tonsil hypertrophy (**E**).

The echocardiography (ECG) revealed ventricular septal hypertrophy, left ventricular hypertrophy, and left ventricular outflow tract obstruction. The dynamic electrocardiogram (DCG) demonstrated sinus arrhythmia, sporadic premature atrial beats, and frequent premature ventricular beats. The cardiac color ultrasound identified the congenital heart disease with obstructive hypertrophic cardiomyopathy and left ventricular outflow tract stenosis. In addition, the cardiac magnetic resonance imaging (MRI) also showed obstructive hypertrophic cardiomyopathy with heterogeneous left ventricular hypertrophy and left ventricular outflow tract jet sign (Figures 2A–D). The brain MRI examination showed the left pretemporal space was slightly wider. Moreover, the thyroid ultrasonography (USG) examination showed a small thyroid gland, while the thyroid hormone was normal. The chest x-ray increased bronchovascular shadows. While, the visual acuity, hearing tests, and abdominal ultrasonography were normal.

Given the special clinical signs she had presented, whole exome sequencing (WES) was performed when she was 3 months old. The result showed a heterozygous X-linked missense variant in the *NAA10* gene [NM_003491: exon5, c. 247C > T, p. (Arg83Cys)], which had been previously reported as pathogenic in variant databases (Clin Var, HGMD) and occurred in a region conserved across species. The Sanger sequencing revealed this variant was not detected in either of her parents, thus arising *de novo* in the proband. In addition, her karyotype was 46, XX.

Then she had since been treated with metoprolol tartrate tablets to control her arrhythmia, with the heart rate peaked at 189 beats per minute. The DCG and ECG were performed regularly to monitor her condition. The results were evaluated by a professional medical team and the medication dosage was adjusted according to the effect of treatment. She also suffered from upper respiratory infections several times during this period, but all had satisfactory outcomes after the treatment.

**Figure 3:**
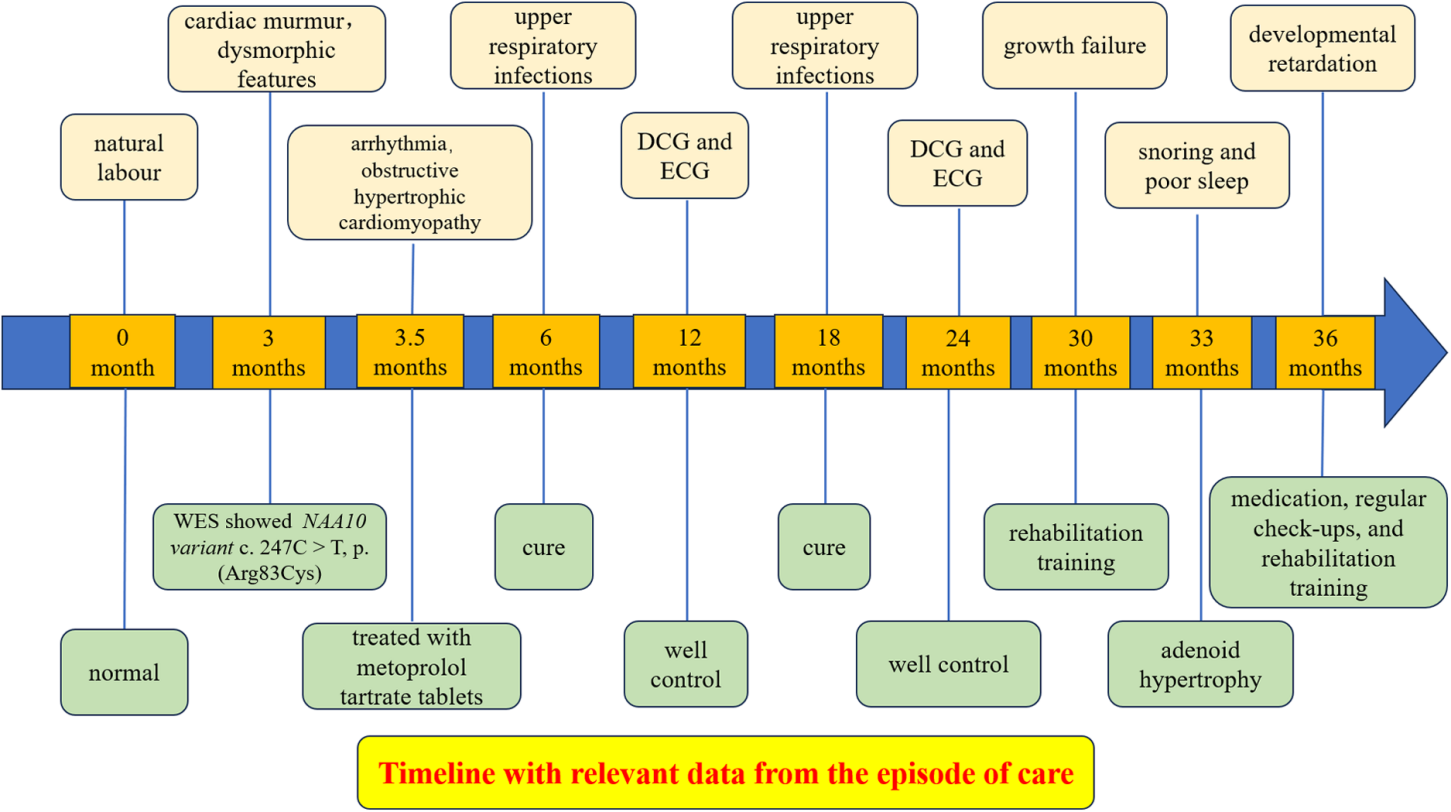
Timeline with relevant data from the episode of care.

Although the arrhythmia and infections were well controlled, her clinical course was complicated by growth failure. The Griffiths Development Scales-Chinese Edition (GDS-C) and the Family Environment Scale Chinese Version (FES-CV) were performed for her at 30 months of age to evaluate her global development. The results showed that the total quotient of GDS-C was 66.71, and the total score of FES-CV was 54. In general, the results indicated that her overall development lagged, and her listening, language, as well as performance abilities, were weak. While the special examination showed that her muscle tone is normal, and her muscle strength level is V. The rehabilitation physician recommended her to do home rehabilitation training and professional rehabilitation training continuously. Moreover, it was worth noting that the result of the Screening Tool for Autism in Toddlers and Young Children (STAT) was high risk. Later, she often snores because of adenoid hypertrophy (Figure 2E), which may also be the cause of her poor sleep. In addition, her last visit was at 34 months for an allergy caused by eating crab, which improved after treatment (Figure 3).

Now she is 3 years old, and her overall development is about half a year behind normal. Recent cardiac color ultrasonography also suggests obstructive hypertrophic cardiomyopathy with moderate mitral regurgitation and mild tricuspid regurgitation. Although her global condition is well yet, medication, regular check-ups, and rehabilitation training under the guidance of a professional medical team are still required for her. In addition, the risk of autism also needs further evaluation or intervention.

## Discussion

With the various *NAA10* variants that have been identified, the phenotypic spectrum of individuals has vastly expanded in recent years. To date, 106 individuals with *NAA10*-related syndrome carrying 34 different *de novo NAA10* missense variants have been reported (14). The *NAA10*-related syndrome is characterized by a broad spectrum of phenotypes in both males and females, ranging from asymptomatic girls to early lethality caused by structural or conductional cardiac abnormalities with the variable variant. The neurodevelopmental defects, including diverse degrees of ID, motor and language disability, autism spectrum disorder (ASD), and behavioral abnormities, were described as the most common features in all patients (15–17). In addition, facial dysmorphism is also universal but with no clear pattern. Moreover, postnatal growth retardation, impaired motor function, hypotonia, abnormal brain imaging, and recurrent infections were described frequently (9, 18). And in particular, cardiovascular defects were common, often leading to severe outcomes and in some cases fatal. However, due to the extremely limited number of clinical cases with each *NAA10* variant, the genotype–phenotype relationship does not appear to be clear yet.

Furthermore, the levels of remaining enzymatic activity and the diverse function of *NAA10* were discussed as the foundation underlying the *NAA10*-associated phenotypes (19–21). Nevertheless, a solid conclusion could not be drawn yet due to the less affected individuals. Functional research of diverse variants has been performed in previous studies, and the NatA complex stability as well as the global NatA acetyltransferase activity have been tested. Studies have shown that p. Val111Gly and p. Arg116Trp variants usually lead to mild phenotypes (6, 15). It is worth noting that different experimental approaches have been used in the functional characterization of NAA10 variants. For instance, Saunier et al. tested the enzymatic activity of purified monomeric NAA10 (not as part of the NatA complex) when studying the impact on Arg83Cys and Arg116Trp (6). When compared to the nearly abolished catalytic activity associated with the p. Phe128Leu, p. Phe128Ile, and p. Val107Phe variants, the partial catalytic impairment (about 60%) of the NAA10 subunit caused by the p. Arg83Cys variant was not reflected in different degrees of clinical phenotypes, suggesting the possibility of a critical threshold of enzyme activity necessary for normal function (6). Interestingly, in subsequent functional studies, Cheng et al. revealed that the *NAA10* p. Arg83Cys variant leads to promoted NatA activity, suggesting that the phenotype of individuals with such variant might express via a particular mechanism (5).

In fact, the p. Arg83Cys variant identified in our patient is the most common variant and has been previously reported as pathogenic and occurs in a region conserved across species (10, 14). The phenotype described in this subtype usually consisted of variable degrees of neurodevelopmental defects, such as moderate to severe ID, motor and language disability, and ASD (5, 6). Feeding difficulties and postnatal failure to thrive with final short stature were described in approximately two-thirds of the patients. Facial dysmorphisms, including a prominent forehead, low-set ears, broad and flat nasal bridge, and particularly coarse face were depicted in more than half of the sufferings, while a specific pattern has not been identified. Furthermore, visual impairment, brain imaging anomalies, hypotonia, visual impairment, and recurrent infections were common too. What calls for special attention is that half of the patients exhibited different degrees of congenital structural cardiac abnormalities or arrhythmia (22). While, skeletal defects, hearing impairment, and seizures were described less frequently (22).

Overall, our patient confirmed the main clinical manifestations described previously such as dysmorphic features, developmental disorders, as well as congenital structural and conditional cardiac abnormalities. What's more, our patient also showed some rare but compatible features, including exophthalmos, blue sclera, and cutaneous capillary malformations. She also suffered from poor sleep, snoring, and facial changes caused by adenoid hypertrophy. The new rare clinical features expand the phenotype of *NAA10*-related syndrome. Her clinical course may enrich the knowledge of the trajectories and prognoses in patients with such conditions. Furthermore, given the fatal cardiac malformations or arrhythmias observed in the previous individuals, we recommend that patients should undergo precise medical follow-up, particularly for cardiac diseases (including hypertrophic cardiomyopathy and arrhythmias). In addition, her autistic tendencies also deserve further research to confirm.

It is well depicted that heterozygous females with *NAA10*-related syndrome have a wide spectrum of clinical manifestations, ranging from asymptomatic to varying degrees of developmental disorders and cardiac defects (6, 13, 22). It depends not only on the activity and stability of NatA acetyltransferase caused by specific variant types but also on X-chromosome skewing. Because most carrier mothers of boys affected with Ogden syndrome were asymptomatic, the X-chromosome inactivation (XCI) was almost completely skewed toward the wild-type allele (21). Given the present inability to speculate the genotype–phenotype correlations based on XCI, sufficient investigations should devote to exploring the effects of XCI on this syndrome to calculate its impact on phenotypic expression.

## Conclusions

In summary, our patient confirmed the most common variant c. 247C > T, p. (Arg83Cys) and the main clinical manifestations such as dysmorphic features, developmental disorders, as well as congenital structural and conductional cardiac abnormalities. She also showed some rare but compatible features, including exophthalmos, blue sclera, adenoid hypertrophy, and cutaneous capillary malformations, which expand the phenotype of *NAA10*-related syndrome.

## Data Availability

The original contributions presented in the study are included in the article, further inquiries can be directed to the corresponding author.
